# New Insights into the Biology, Ecology, and Management of Mosquitoes

**DOI:** 10.3390/insects16060577

**Published:** 2025-05-29

**Authors:** Athanasios Giatropoulos

**Affiliations:** Laboratory of Efficacy Control of Pesticides, Benaki Phytopathological Institute, 14561 Kifissia, Greece; a.giatropoulos@bpi.gr

Mosquitoes pose a great threat to human and animal health as vectors of many important diseases such as malaria, dengue, West Nile virus, yellow fever, filariasis, and encephalitis. The successful management of mosquitoes requires not only an integrated mosquito management concept, in which all appropriate methods for control are used, but also knowledge of the biology and ecology of the target organisms [1]. In this Special Issue, fourteen original research articles aim to highlight recent advances in the knowledge on the mosquito life-cycle characteristics affected by competitive interactions between mosquitoes, as well as on the distribution and abundance of mosquito species. This Special Issue also reports recent advances in integrated mosquito management, including surveillance methods, the use of synthetic insecticides and repellents, botanicals, sterile insect technique, and insecticide resistance management. The research areas covered by the scope of this Special Issue include medical and veterinary entomology, applied entomology, parasitology, mosquito bio-ecology, and mosquito control.

The co-existence of different mosquito species in breeding sites may lead to interspecific competition at the larval stage for limited resources such as food and space. Competitive interactions may affect mosquitoes’ larval developmental rate, survival, and behaviors, such as predation and cannibalism, thus influencing the species composition and abundance in the ecosystem [2,3,4]. Lushasi et al. (Contribution 1) investigated the effects of larval competition between *Aedes aegypti* and *Anopheles arabiensis*, *Anopheles gambiae* s.s., or *Anopheles funestus* under semi-field conditions. The results revealed that the interspecific competition remarkably affected both tested genera; however, the effects on *Anopheles* species were more profound compared to on *Ae. aegypti*. These interactions may have consequences in the transmission of diseases and serve as biological markers to indicate the effects of vector control measures.

The implementation of effective mosquito management programs relies on entomological surveillance by collecting data on mosquito species composition and abundance [5]. The study by Bisia et al. (Contribution 2) is about tracking different types of mosquitoes and the spread of the invasive Asian tiger mosquito (*Aedes albopictus*) on the remote Greek island of Kastellorizo. They conducted KAP (knowledge, attitude, and practices) surveys, set up mosquito traps, and identified the types of mosquitoes found. The study revealed the presence of *Ae. albopictus* and other species like *Culex pipiens*. It was concluded that the involvement of the community in mosquito monitoring is critical for the management of the mosquito population. Bisia et al. (Contribution 3) explored the distribution of mosquito species in the Attica region of Greece for two years, using traps across various locations. The mosquito caches in the traps showed that *Ae. albopictus* and *Cx. pipiens* s.l. are widespread in the region. In some cases, mosquito species were confirmed via DNA. Differences in the distribution of mosquito species among different locations throughout the season were found, underscoring the necessity for continuous monitoring and control measures to protect public health. The work by Paiz-Moscoso et al. (Contribution 4) aimed to develop and assess an adhesive lure trap for the surveillance of *Aedes aegypti* indoors. They considered four compounds with remarkable attraction to *Ae*. *aegypti* and a blend determined through preliminary laboratory assays. A low-cost, sticky cardboard trap was developed incorporating the selected blend. Its effectiveness was tested in semi-field and field trials. The attractance of the trap was comparable with the gold-standard BG-Sentinel trap during the field evaluation in an indoor house environment.

The sterile insect technique (SIT) is a novel and eco-friendly mosquito management tool that can suppress mosquito populations [6]. The study by Hapugoda et al. (Contribution 5) aimed to evaluate the performance of sterile males with respect to the wild male population in a Mark–Release–Recapture (MRR) field trial in Sri Lanka. The results revealed that the flight distance of sterile males was up to 543.8 m, with a mean distance of 255.1 ± 44.6 m, and that they survived a maximum of 6 days, with a mean life span of 3.55 ± 2.32 days. The sterile males exerted high mating competitiveness in the field based on the Fried value estimated (0.47) and caused remarkable induced sterility in the wild eggs in the second generation. Balatsos et al. (Contribution 6) studied the longevity and frailty of sterile, non-sterile, and wild male *Ae. albopictus* mosquitoes, using a novel captive cohort method for the SIT, according to the IAEA protocol. The results revealed that marking male mosquitoes does not affect their longevity under controlled conditions, and that sterilization had no negative impact on male longevity. Additionally, the released sterile males showed increased longevity compared to non-sterile males. These results indicate the necessity of ongoing research for the optimization of mass rearing, sterilization, and transportation methods for sterile males to be used in SIT programs. The research by Wang et al. (Contribution 7) was aimed at the potential use of the Varian Clinac 23EX linear accelerator, typically applied in X-ray cancer treatment, as an alternative irradiation source for the sterilization of male mosquitoes to be used in SIT mosquito control programs. Their findings showed that the adjustment of the X-ray dosage to a specific level may result in a sterilization effect, similar to that achieved using traditional γ-ray sources.

Personal protection from mosquito blood feeding mostly consists of treating clothing with insecticides and the use of repellents on clothing and the skin, while other technologies using a high voltage circuit or voltage booster to kill or repel mosquitoes have been used [7]. Luan et al. (Contribution 8) developed a low-voltage, mosquito-resistant cloth (MRC) against *Ae. aegypti* that blocked blood feeding across the textile and was flexible and breathable. The design was based on mosquito head and proboscis morphometrics, the development of a novel 3D textile with the outer conductive layers insulated from each other with an inner, non-conductive woven mesh, and the use of a DC (direct current; extra-low-voltage) resistor–capacitor. Their results demonstrated for the first time the use of biomimetic mosquito-repelling technology to prevent blood feeding using extra-low energy consumption.

Botanical insecticides and their components are regarded as important alternative management tools against mosquitoes considering mosquito resistance issues and the negative side effects of the excessive use of synthetic chemicals on the environment and human health [8]. Monoterpenes are major components in many botanicals, such as essential oils and extracts, that can be used as novel, effective, and environmentally friendly alternative mosquito control agents [9]. Sittichoket al. (Contribution 9) studied the larvicidal and pupicidal properties of two monoterpenes—geranial and *trans*-cinnamaldehyde—and their combinations on *Ae. aegypti*. Binary mixtures of the tested botanical formulations were remarkably more effective than single formulations or the reference formulation (temephos). Also, these mixtures did not affect the non-target aquatic predator, guppies (*Poecilia reticulata*). In the study by Farina et al. (Contribution 10) the leaves and flowers of *Sambucus ebulus* were extracted in methanol and water and the crude extracts were chemically characterized. The extracts and some of their components (phenolics and amino acids) were tested as larvicides against *Ae. albopictus* and *Cx. pipiens*. The most potent larvicide against *Ae. albopictus* after 24 h was gallic acid, followed by the crude *S. ebulus* leaf extract. The most toxic compound on *Cx. pipiens* larvae was the crude flower extract. In both mosquito species, the leaf extract caused higher AChE inhibition than the flower extract. Moreover, the leaf extract resulted in oviposition deterrence on *Ae. albopictus* females from the third day.

The microbiota found in mosquito breeding sites can affect mosquito ovipositioning and larval development, change the bacterial composition in the gut of mosquito adults, and impact the transmission of pathogens such as malaria parasites [10]. Using DNA extraction, Castañeda-Espinosa et al. (Contribution 11) characterized the composition of microorganisms in water from artificial mosquito breeding sites, immature stages, and adults of *Ae. aegypti* in Leticia, Amazonas, Colombia. The results revealed a plethora of bacteria genera in breeding sites and the developmental stages of *Ae. aegypti*. Some of these bacteria may have potential biotechnological, entomopathogenic, and antiviral properties. Moreover, the study indicated a close relationship between the bacterial composition in the water body of breeding sites and the physicochemical characteristics of the different types of breeding sites. Duque-Granda et al. (Contribution 12) investigated the bacterial communities in two *Anopheles* species collected from Leticia, Amazonas, Colombia during the dry season, using DNA extraction and sequencing. The findings revealed that the bacterial communities differed between mosquito species. Specific genera of bacteria are reported as inhibitors of parasite development or beneficial for the development of mosquitoes. The results of the study underscore the potential of these microorganisms as agents for biotechnological interventions aiming at mosquito control, and the necessity of further investigating the specific role of these bacteria in mosquito species.

Several studies have shown changes the in bacterial composition associated with mosquito insecticide resistance [11,12,13]. The study by Viafara-Campo et al. (Contribution 13) evaluated the resistance of *Ae. aegypti* larvae to temephos insecticide and adult females to deltamethrin in populations from Caquetá, Colombia. It also characterized the intestinal bacteria in these mosquitoes. According to the results, there was a difference in the culturable bacteria found in deltamethrin-resistant females and those present in untreated females and temephos-resistant larvae. This study indicates that gut microbiota may be involved in insecticide resistance, providing valuable insights for understanding new resistance mechanisms.

Recent studies have highlighted the role of miRNAs in insecticide response and resistance mechanisms, showing that their differential expression can influence key detoxification genes, such as cytochrome P450s (CYPs), glutathione S-transferases (GSTs), and esterases [14]. Trujillo-Rodríguez et al. (Contribution 14) analyzed miRNA expression in *Ae. aegypti* following imidacloprid exposure, comparing a field strain and a susceptible reference strain. After exposing these mosquitoes to imidacloprid (1 µg/mL), they assessed changes in the microRNA expression. Their results identified microRNAs present in all mosquitoes, and others only expressed in wild mosquitoes following insecticide exposure. These findings suggest that specific microRNAs may be associated with insecticide response and potentially linked to resistance mechanisms.

Overall, the main outcomes of the research articles presented herein are outlined as follows:

- Ιn semi-field conditions, interspecific larval competition between *Ae. aegypti* and *Anopheles arabiensis*, *An. gambiae* s.s., or *An. funestus* remarkably affected both tested genera; however, the effects on *Anopheles* species were more profound compared to on *Ae. aegypti* (Contribution 1);
- Mosquito surveillance in Kastellorizo (Greek island), revealed the presence of *Ae. albopictus, Aedes cretinus*, and *Cx. pipiens*, and indicated the necessity of community awareness and education for the effective management of mosquito populations (Contribution 2);
- Mosquito surveillance for two years in the Attica region of Greece revealed the widespread distribution of *Ae. albopictus* and *Cx. pipiens* s.l. in the region, indicating the differences in the distribution of mosquito species across different locations throughout the season (Contribution 3);
- The development of a low-cost attractant sticky trap for the surveillance of *Ae. aegypti* populations indoors (Contribution 4);
- The performance of gamma-ray-sterilized males was evaluated in a Mark–Release–Recapture field trial conducted in Sri Lanka against *Ae. albopictus* to be used in future SIT trials (Contribution 5);
- The captive cohort method was used for the first time to assess the biological dynamics of sterile mosquitoes in SIT programs, underscoring the necessity of continuous research to optimize mass rearing, sterilization, and transportation methods for the production of sterile males to be used in SIT programs (Contribution 6);
- The X-ray machine typically employed in cancer treatment can be applied as an alternative radiation source for the sterilization of male mosquitoes to be used in SIT mosquito control programs (Contribution 7);
- A novel 3D textile was developed based on the mosquito head structure of *Ae. aegypti*; when charged with 15 volts, it was 100% effective in preventing mosquito blood feeding across an artificial membrane (Contribution 8);
- Binary mixtures of two monoterpenes, namely geranial and *trans*-cinnamaldehyde, were more effective than single formulations or temephos against the larvae of *Ae. aegypti*, and did not affect the non-target aquatic predator, guppies (*Poecilia reticulata*) (Contribution 9);
- In laboratory bioassays, the larvicidal properties of the crude *S. ebulus* leaf extract and gallic acid against *Ae. albopictus* and the crude *S. ebulus* flower extract against *Cx. pipiens* were identified (Contribution 10);
- In Leticia, Amazonas, Colombia, a plethora of bacteria genera were found in breeding sites and the developmental stages of *Ae. aegypti,* and a close relationship was identified between the bacterial composition in the water body of breeding sites and the physicochemical characteristics of the different types of breeding sites (Contribution 11);
- In Leticia, Amazonas, Colombia, a high richness in bacterial diversity was recorded across different mosquito life stages in two *Anopheles* species, underscoring the potential of these microorganisms as agents for biotechnological interventions aiming at mosquito control (Contribution 12);
- The gut microbiota may be involved in the insecticide resistance of *Ae. aegypti* to temephos and deltamethrin (Contribution 13);
- After exposing *Ae. aegypti* mosquitoes to imidacloprid, some microRNAs were expressed in wild mosquitoes following insecticide exposure, suggesting that specific microRNAs may be associated with insecticide response and potentially linked to resistance mechanisms (Contribution 14).

It is my hope that these publications, which refresh our current knowledge on the biology, ecology, and management of mosquitoes, will inform and trigger future research and efforts to better understand aspects related to mosquito bio-ecology and develop novel and sustainable mosquito management strategies.

## References

[1] Becker N., Petrić D., Zgomba M., Boase C., Madon M.B., Dahl C., Kaiser A. (2020). Mosquitoes: Identification, Ecology and Control.

[2] Giatropoulos A., Papachristos D., Michaelakis A., Kapranas A., Emmanouel N. (2022). Laboratory Study on Larval Competition between Two Related Mosquito Species: *Aedes* (*Stegomyia*) *albopictus* and *Aedes* (*Stegomyia*) *cretinus*. Acta Trop..

[3] Tabachnick W.J. (2013). Nature, nurture and evolution of intra-species variation in mosquito arbovirus transmission competence. Int. J. Environ. Res. Public Health.

[4] Muturi E.J., Kim C.H., Jacob B., Murphy S., Novak R.J. (2010). Interspecies predation between *Anopheles gambiae* s.s. and *Culex quinquefasciatus* larvae. J. Med. Entomol..

[5] Hu W., Clements A., Williams G., Tong S. (2010). Dengue fever and El Nino/Southern Oscillation in Queensland, Australia: A time series predictive model. Occup. Environ. Med..

[6] Lees R.S., Gilles J.R., Hendrichs J., Vreysen M.J., Bourtzis K. (2015). Back to the future: The sterile insect technique against mosquito disease vectors. Curr. Opin. Insect Sci..

[7] Gordon U., Tanveer F., Rose A., Paaijmans K., Corona C., Debboun M., Coats J. (2022). CHAPTER 6—Repelling mosquitoes with electric fields. Advances in Arthropod Repellents.

[8] Ntalli N., Koliopoulos G., Giatropoulos A., Menkissoglu-Spiroudi U. (2019). Plant secondary metabolites against arthropods of medical importance. Phytochem. Rev..

[9] Giatropoulos A., Koliopoulos G., Pantelakis P.-N., Papachristos D., Michaelakis A. (2023). Evaluating the Sublethal Effects of *Origanum vulgare* Essential Oil and Carvacrol on the Biological Characteristics of *Culex pipiens* biotype molestus (Diptera: Culicidae). Insects.

[10] Nilsson L.K.J., de Oliveira M.R., Marinotti O., Rocha E.M., Håkansson S., Tadei W.P., de Souza A.Q.L., Terenius O. (2019). Characterization of Bacterial Communities in Breeding Waters of Anopheles Darlingi in Manaus in the Amazon Basin Malaria-Endemic Area. Microb. Ecol..

[11] Dada N., Sheth M., Liebman K., Pinto J., Lenhart A. (2018). Whole Metagenome Sequencing Reveals Links between Mosquito Microbiota and Insecticide Resistance in Malaria Vectors. Sci. Rep..

[12] Arévalo-Cortés A., Mejia-Jaramillo A.M., Granada Y., Coatsworth H., Lowenberger C., Triana-Chavez O. (2020). The Midgut Microbiota of Colombian *Aedes aegypti* Populations with Different Levels of Resistance to the Insecticide Lambda-Cyhalothrin. Insects.

[13] Wang H., Zhang C., Cheng P., Wang Y., Liu H., Wang H., Wang H., Gong M. (2021). Differences in the Intestinal Microbiota between Insecticide-Resistant and -Sensitive *Aedes albopictus* Based on Full-Length 16S RRNA Sequencing. Microbiologyopen.

[14] Qiao J., Du Y., Yu J., Guo J. (2019). MicroRNAs as Potential Biomarkers of Insecticide Exposure: A Review. Chem. Res. Toxicol..

